# Gout, hyperuricemia, and male reproductive health: a systematic review and meta-analysis

**DOI:** 10.3389/fimmu.2026.1889720

**Published:** 2026-09-15

**Authors:** Jiping Mao, Mangmang Xu, Yanfang Tang, Jiaqi Chen, Jia Zhou, Dehong Wu

**Affiliations:** 1 The Second Clinical Medical College of Zhejiang Chinese Medical University, Hangzhou, China; 2 Department of Rheumatology, The Second Affiliated Hospital of Zhejiang Chinese Medical University (Xinhua Hospital of Zhejiang Province), Hangzhou, China; 3 School of Basic Medical Sciences, Zhejiang Chinese Medical University, Hangzhou, China

**Keywords:** erectile dysfunction, gout, hyperuricemia, infertility, inflammation, male, reproductive hormones

## Abstract

**Background:**

Gout is an inflammatory arthritis characterized by hyperuricemia in humans. Male patients with gout or hyperuricemia have a wide range of reproductive health challenges. However, research on the sexual and reproductive health of male patients with gout or hyperuricemia remains limited.

**Objective:**

This systematic review and meta-analysis investigated the impact of gout and/or hyperuricemia and its associated factors on male sexual and reproductive health.

**Methods:**

The PubMed, Cochrane, Embase, Web of Science, Wanfang Data, and China National Knowledge Infrastructure (CNKI) databases were searched for relevant articles from January 2000 to October 2025. Two reviewers extracted the data and completed quality assessment using the Newcastle-Ottawa Scale and the Agency for Healthcare Research and Quality checklist. Meta-analysis was conducted using Review Manager 5.4 and Stata 18.0. The protocol was registered on PROSPERO (CRD 420261302205). The outcomes such as sexual function, reproductive hormones, and fertility were evaluated.

**Results:**

In the literature search, 1,527 articles were retrieved. Among these, 11 studies met the inclusion criteria. This meta-analysis included 104,277 patients with gout or hyperuricemia and 342,621 control subjects. The meta-analysis results showed that the risk of erectile dysfunction was higher in patients with gout (RR = 1.37; 95% CI = 1.14 to 1.65, *P* < 0.001). Compared with the control group, patients with gout or hyperuricemia showed lower levels of testosterone and estradiol (testosterone: MD = −1.00, 95% CI = −1.39 to −0.62, *P* < 0.001; estradiol: MD = −4.67, 95% CI = −9.31 to −0.02, *P* = 0.05). A single study showed that semen volume and total sperm counts were lower among infertile men in the hyperuricemia group.

**Conclusion:**

Male patients with gout or hyperuricemia are more prone to erectile dysfunction and lower levels of reproductive hormones. Patients with gout showed significantly lower testosterone levels in the subgroup analyses. These findings emphasize the clinical need for increased awareness of male reproductive health problems in men with gout and implementation of effective intervention measures.

**Systematic review registration:**

https://www.crd.york.ac.uk/PROSPERO/, identifier CRD420261302205.

## 1 Introduction

Gout is a clinical syndrome characterized by hyperuricemia, which is caused by purine metabolism disorders or impaired renal uric acid excretion. It manifests as acute or chronic inflammation and tissue damage due to urate crystal deposition in tissues and organs (1). It is the most common inflammatory arthritis in humans and significantly impacts healthcare costs, productivity, quality of life, and physical activity. Reports on the prevalence and incidence of gout vary significantly depending on the study population and methodology employed. The prevalence rates range from < 1% to 6.8% and the incidence rates range between 0.58 and 2.89 per 1,000 person-years (2). Moreover, patients with gout are at a higher risk of developing comorbidities than the general population, including chronic kidney disease, hypertension, cardiovascular disease, metabolic syndrome, and diabetes (3–5).

Hyperuricemia is defined as serum uric acid levels exceeding 420 μmol/L on two non-consecutive days (6). It is more prevalent than gout, affecting approximately 15% to 25% of adult men, and is increasing rapidly among those of reproductive age (7, 8). Even in the absence of gouty arthritis, hyperuricemia is independently associated with metabolic syndrome, diabetes, hypertension, and cardiovascular disease (4, 9, 10). This highlights the importance of examining gout and hyperuricemia separately because they may have different effects on male reproductive health.

Globally, infertility affects 8-12% of couples in the reproductive age, with male factors accounting for approximately 50% of cases (11). The causes of impaired male fertility include varicocele, hypogonadism, Klinefelter syndrome, genetic disorders, or oxidative stress-related infertility (12). Furthermore, endocrine dysfunction, autoimmune diseases, chronic kidney disease, diabetes, exposure to reproductive toxins, and various lifestyle factors such as smoking, excessive alcohol consumption, recreational drug use, obesity, and psychological stress are considered as potential risk factors for male infertility (13, 14). Therefore, multiple complications and risk factors related to gout or hyperuricemia may impact the reproductive health of male patients with gout or hyperuricemia, meaning they may face a wide range of reproductive health challenges, including sexual dysfunction, reproductive hormone imbalances, and impaired semen quality.

Accumulating evidence suggests that male patients with gout or hyperuricemia face reproductive and sexual health challenges. The inflammation and vascular endothelial damage associated with gout may contribute to erectile dysfunction (ED) (15). A study by Sultan et al. involving 9,653 male gout patients reported that 1,736 patients (18%) experienced varying degrees of sexual dysfunction (16). Furthermore, patients with gout or hyperuricemia show abnormal levels of reproductive hormones, including testosterone (T), estradiol (E_2_), luteinizing hormone (LH), follicle-stimulating hormone (FSH), and prolactin (PRL) (17–22). Although evidence is limited to a single trial in infertile men, semen quality, especially sperm count and motility, is adversely affected in men with hyperuricemia (19, 23). Studies on male fertility in patients with gout, based on urological evaluations, hormonal profiling, and semen analysis, have suggested potential associations between these conditions and adverse reproductive health outcomes. However, these assessments do not represent comprehensive evaluation of the male reproductive potential. Moreover, comprehensive systematic examination of male reproductive capacity in the context of gout or hyperuricemia has not yet been conducted.

Meanwhile, a study based on the NHANES database reported that approximately one-third of patients with gout received urate-lowering therapy (ULT) in the form of treatment with allopurinol and febuxostat (24). However, the benefits of ULT on the male reproductive health remains controversial.

Because of social, family, and personal physiological and psychological needs, young and middle-aged men of reproductive age with gout or hyperuricemia are concerned about fertility during medical consultations. Rheumatologists and primary care physicians should therefore pay close attention to the reproductive health of these patients. This systematic review and meta-analysis aims to consolidate currently available evidence regarding the impact of gout and hyperuricemia on male reproductive health and to provide valuable suggestions for clinicians managing patients with uric acid-related disorders.

## 2 Materials and methods

The research protocol for this systematic review and meta-analysis followed the Preferred Reporting Items for Systematic Reviews and Meta-Analyses 2020 (PRISMA 2020) guidelines (25). This review was registered in PROSPERO (International Prospective Register of Systematic Reviews (Registration number: CRD 420261302205) (**Supplementary File 1**) to ensure transparency and adherence to predefined objectives and methods.

### 2.1 Data source and search strategy

We conducted a systematic search of the PubMed, Cochrane, Embase, Web of Science, CNKI, and Wanfang databases from January 2000 to October 2025 to identify eligible studies without any language restrictions. Medical Subject Headings (MeSH) terms and free-text keywords were used based on the specifications of each database. The search terms included MeSH terms and free-text keywords related to the following three domains: (1) the population (“infertility, male” OR “male reproductive health”); (2) the exposure (“hyperuricemia” OR “gout”); and (3) the outcomes (“sexual dysfunction” OR “erectile dysfunction” OR “testosterone” OR “estradiol” OR “sperm quality” OR “semen parameters” OR “fertility”). The entire search strategy for each database is provided in **Supplementary File 2**. Furthermore, we reviewed the reference lists of retrieved articles to identify further relevant studies.

### 2.2 Outcomes

The primary outcome of this study was to assess the reproductive health of male patients with gout or hyperuricemia in the following three dimensions: (1) Sexual function [sexual dysfunction (SD), erectile dysfunction (ED)]; (2) Reproductive hormones (T and E_2_); (3) Fertility (sperm quality, sperm count, etc.).

### 2.3 Inclusion and exclusion criteria

The inclusion criteria were as follows: (1) Cross-sectional studies, cohort studies, and case-control studies with more than 10 cases; (2) Men diagnosed with gout or hyperuricemia according to any diagnostic criteria (26–28); (3) Relative risk (RR) estimates or odds ratios (ORs) with 95% confidence intervals (CIs) were provided in the included studies or calculated from the main data; (4) studies including outcomes related to reproductive health.

Exclusion criteria were as follows: (1) Reviews, editorials, letters, conference abstracts, as well as reviews or meta-analyses without original studies; (2) Studies from which valid outcome data could not be extracted or that did not provide comparative information; (3) Duplicate studies, animal experiments, or *in vitro* studies; (4) Studies without full-text access.

### 2.4 Data extraction and quality assessment

Two reviewers (J.P. Mao and M.M. Xu) independently screened literature according to the predefined search strategy and identified eligible articles. Then, they independently extracted data from these selected articles to create tables that included the primary author, publication year, study type, mean age of participants, diagnostic criteria for gout, sample sizes of cases and controls, baseline characteristics of the study population, and male reproductive health outcomes. Any disagreements between reviewers were resolved by discussion until consensus was reached. When necessary, the third reviewer (Y.F. Tang) was consulted to ensure objectivity and accuracy. When detailed data could not be extracted from a study, the corresponding authors were contacted via email to obtain further information.

The methodological quality of included studies was independently assessed by two reviewers using appropriate quality assessment tools and compared the results. The Newcastle-Ottawa Scale (NOS) was used to assess the risk of bias in cohort studies (29), and studies scoring 7 to 9 points were considered high quality. The methodological quality of cross-sectional studies was assessed using the Agency for Healthcare Research and Quality (AHRQ) checklist (30). This checklist evaluated risk of bias across the following five domains: selection bias, performance bias, follow-up bias, measurement bias, and reporting bias. Items were judged as low risk, high risk, or unclear. Studies with scores between 8 to 11 were considered high quality. In cases of disagreement, a third reviewer was consulted.

To minimize errors in data extraction from non-English articles, we implemented the following strategies (1): independent extraction by two reviewers (J.P. Mao and M.M. Xu); (2) use standardized data forms to minimize reliance on narrative interpretation; (3) for research studies published in non-native language, we used professional translation tools and consulted with fluent colleagues to supplement; and (4) cross-validate key data against original tables or figures and contact authors when ambiguity persists.

### 2.5 Statistical analysis

We performed meta-analyses of outcomes with sufficient data (more than two studies providing valid data) using Review Manager 5.4 and Stata 18.0 software. Binary outcomes were presented using RR and 95% CI, whereas continuous outcomes were presented using the mean difference (MD). Heterogeneity was assessed with I² > 50% indicating significant heterogeneity. The random-effects model was used when significant heterogeneity was present (I² > 50%, *P* < 0.05), and the fixed-effects model was used otherwise. *P*-value < 0.05 was considered statistically significant. Sensitivity and subgroup analyses were used to evaluate sources of heterogeneity. Furthermore, outcomes with insufficient data were systematically reviewed. The Begg and Egger tests were conducted using Stata 18.0 to assess publication bias (31, 32).

### 2.6 Subgroup analysis

Subgroup stratification was performed based on age, region, study design, diagnostic criteria, and inflammation. Participants were divided into three age groups (≤34, 35–64, and ≥65 years). The youngest group represented peak male fertility; the middle group corresponded to a high prevalence of ED, gout, and related comorbidities; and the oldest group reflected age-related reproductive decline with low fertility demand. This age categorization was consistent with established epidemiological classifications and ensures sufficient sample sizes for stable subgroup analyses because of the limited number of included studies. Inflammation status was defined operationally by clinical diagnosis. The inflammatory group comprised studies enrolling patients with confirmed gout (ACR/EULAR or ICD criteria), whereas the non-inflammatory group comprised studies enrolling participants with asymptomatic hyperuricemia (serum uric acid >420 μmol/L without gouty arthritis). This classification was based on clinical diagnosis as a proxy for inflammation rather than direct biomarker measurements (e.g., CRP, IL-6) because these parameters were not uniformly reported across studies. Additional subgroups included region, study type, and diagnostic criteria. For details, please refer to the Results - Subgroup Analysis section below.

## 3 Results

### 3.1 Literature retrieval and characteristics

A total of 1,527 articles were retrieved from various databases in the initial search and imported into EndNote. After deleting 273 duplicates, 1,254 articles remained. After a preliminary review of titles and abstracts, 1,183 studies that did not meet the selection criteria were excluded. After full text reading of the remaining 71 studies, we excluded 60 additional articles. Finally, 11 studies were included in this systematic review and meta-analysis (5, 16–22, 33–35). The screening process is shown in **Figure 1**. The main characteristics of all included studies are shown in **Table 1**. The articles 1 to 8 were focused on clinical patients with gout and articles 9 to 11 were studies regarding patients with asymptomatic hyperuricemia. Throughout the results section, we use the term “patients with gout or hyperuricemia” when referring to pooled analyses and explicitly distinguish between the two populations in the subgroup analyses when applicable. **Supplementary Table 1** includes specific data results related to reproductive health in each study).

**Figure 1:**
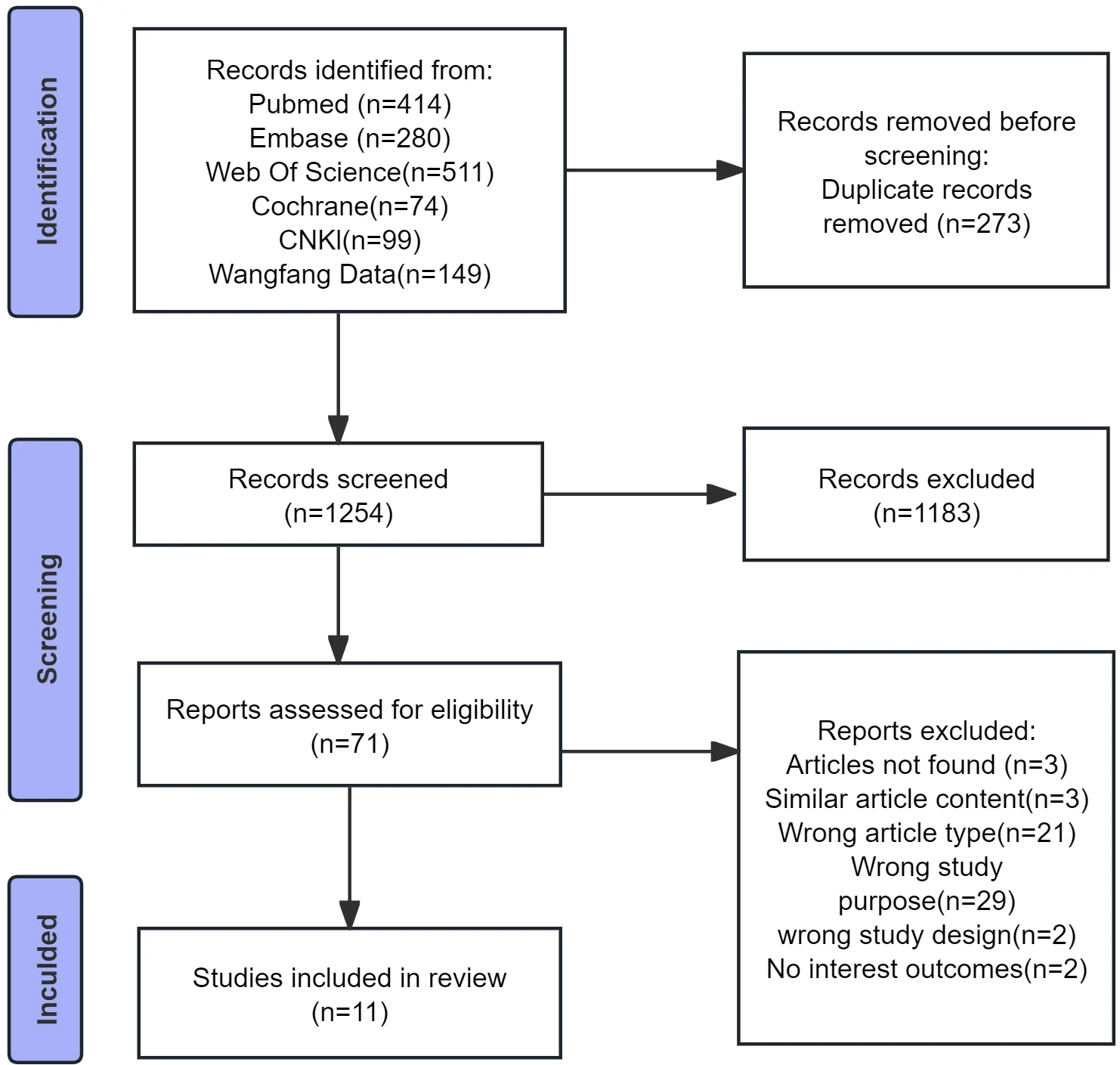
Flow chart of study selection strategy in the meta-analysis.

**Table 1:** The characteristics of studies.

No	Included studies	Year	Area	Study type	Cases		Age (years)		Gout/HUA assessment criteria	Variable adjustment	Outcomes
					Gout/HUA	Control	Gout/HUA	Control			
1	Chen et al. (5)	2015	Taiwan	cohort study	19368	77472	47.2 ± 12	47.2 ± 12	ICD-9-CM Code 274	Age, congestive heart disease, ischemic heart disease, hypertension, depression, and chronic renal failure	①
2	Hsu et al. (34)	2015	Taiwan	cohort study	35265	70529	49.6 ± 16.2	49.1 ± 16.5	ICD-9-CM Code 274	Age, coronary artery disease, peripheral arterial disease, chronic kidney disease, hypertension, diabetes, hyperlipidemia, depression, and anxiety	①
3	Kim et al. (22)	2019	Korean	cross-sectional study	80	70	52 (44–59)	50(42-55)	ACR/MSU	Age, waist circumference, hypertension, and dyslipidemia	①②
4	Schlesinger et al. (35)	2015	England	cross-sectional study	83	118	56.7 ± 14.3	53.52 ± 13.7	Tophus/ clinical features	Age, depression, diabetes, fasting glucose, hypertension, elevated cholesterol level, prostate disease, and heart disease	①
5	Schlesinger et al. (33)	2018	England	cohort study	38438	154332	63.6 ± 12.3	63.6 ± 12.2	Read codes	Age, BMI, smoking, and alcohol consumption, ischemic heart disease, hypertension, hyperlipidemia, and medication use	①
6	Sultan et al. (16)	2017	England	cohort study	9653	38218	18-64	18-64	Read codes	Age, BMI, smoking status, and alcohol consumption, and ischemic heart disease, hypertension, diabetes mellitus, depression, and chronic renal disease	①
7	Yigit et al. (20)	2024	Türkiye	cross-sectional study	134	104	56(48-62)	47(40.5-54.5)	Tophus/ clinical features	Age, BMI, hypertension, hyperlipidemia, insulin resistance, fasting blood glucose, BUN, creatinine, total cholesterol, triglycerides,	①②
8	Li ML et al. (17)	2020	China	cross-sectional study	266	129	44 ± 12	43 ± 11	ACR/EULAR	Age, Triglyceride, BUN, Fasting blood glucose, E_2_, Testosterone, Prog	②
9	Jiang Y et al. (18)	2014	China	cross-sectional study	43	43	35.2 ± 6.1	36.8 ± 7.4	UA ≥420 μmol/L	FSH, LH, PRL, E_2_, Testosterone, Prog, Total cholesterol, Triglyceride	②
10	Ma J et al. (19)	2022	China	cross-sectional study	263	391	28.9 ± 4.3	29.3 ± 4.9	UA ≥420 μmol/L	Age, BMI, FBG, TC, TG, HDL, LDL, SV, SC, TSC, PR, T, FSH, LH, E_2_, PRL	②③
11	Tsai et al. (21)	2022	Taiwan	cohort study	684	1215	44.57 ± 11.22	46.25 ± 11.22	UA ≥420 μmol/L	Age, BMI, HDL, LDL, TG, FBG, T, Hypertension, Diabetes, Hyperlipidemia, Creatinine, Chol	②

ACR, American College of Rheumatology; EULAR, European League Against Rheumatism; HUA, hyperuricemia; ICD, International Classification of Diseases; Sexual health outcomes were classified in 3 categories: ①Sexual function (sexual dysfunction (SD), ED), ②Reproductive hormones (T, LH, FSH, PRL, E_2_), ③Fertility (sperm quality, testicular volume, etc).

### 3.2 Literature quality

The quality assessment results for five cohort studies (5, 16, 21, 33, 34) included in this meta-analysis were based on independent evaluations by two reviewers and are presented in **Supplementary Table 2**. These five studies were rated as high quality. The methodological quality assessment results of the cross-sectional studies included in this meta-analysis are shown in **Supplementary Table 3**. Among these, three studies (17–19) were of moderate quality, and three studies (20, 22, 35) were of high quality.

### 3.3 Outcome analysis

In this meta-analysis, seven articles addressed sexual function (5, 16, 20, 22, 33–35) and were analyzed using the random-effects model. Patients with gout showed significantly increased risk of ED compared with the normouricemic controls (RR = 1.37; 95% CI = 1.14 to 1.65, *P* < 0.001) (**Figure 2**).

**Figure 2:**
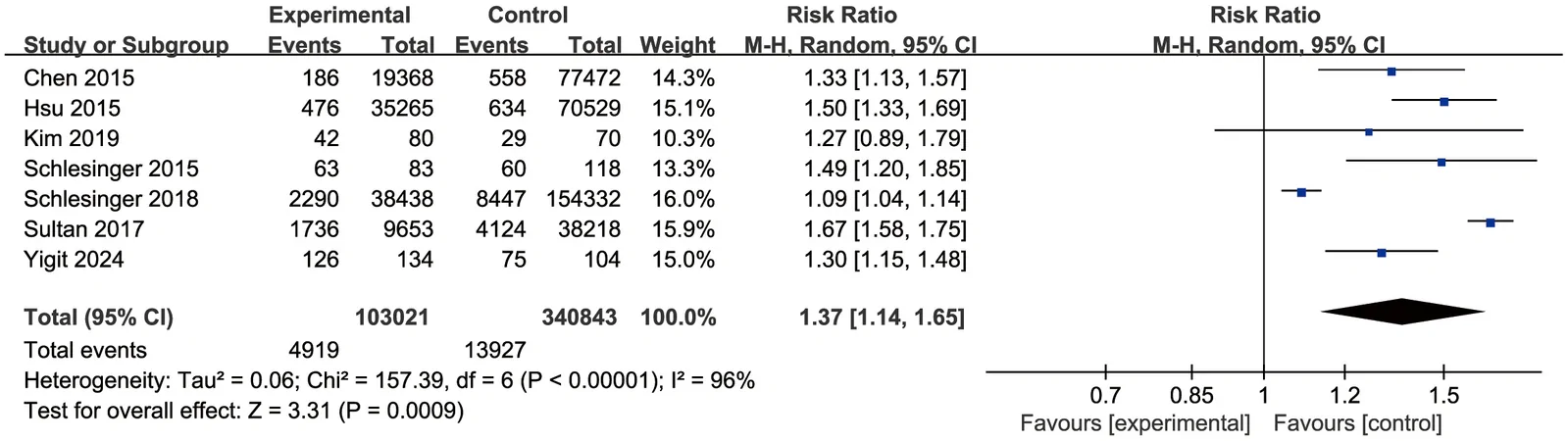
The incidence of ED was higher in patients with gout than in the healthy controls.

A total of six articles (17–22) reported differences in reproductive hormone levels between patients with gout or hyperuricemia and men with normal uric acid levels. We conducted a meta-analysis for hormones with two or more reports. As shown in **Figure 3**, compared with healthy controls, patients with gout or hyperuricemia exhibited decreased trends in T (17–22) and E_2_ (17–19) levels (T: MD = −1.00, 95% CI = −1.39 to −0.62, *P* < 0.001; E_2_: MD = −4.67, 95% CI = −9.31 to −0.02, *P* = 0.05 RE model).

**Figure 3:**
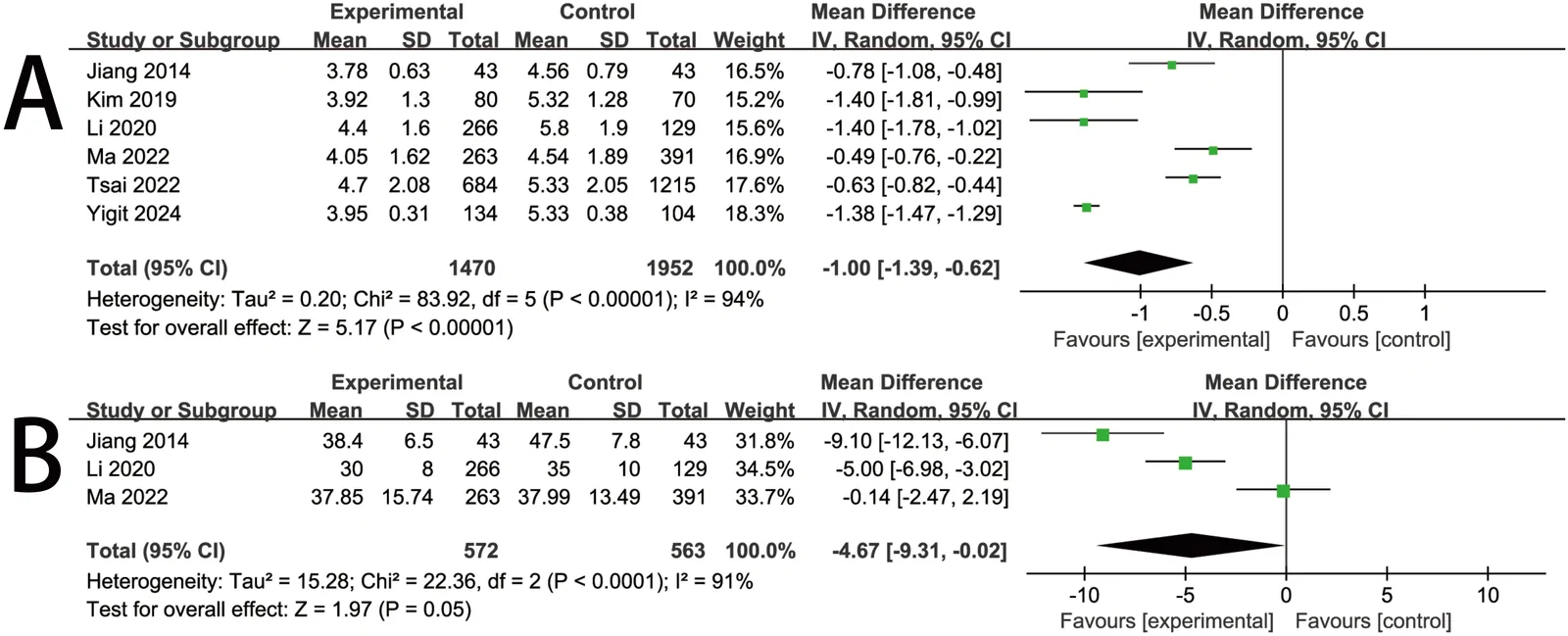
Forest plots of reproductive hormones in patients with gout or hyperuricemia and normouricemic males: **(A)** Testosterone (T); **(B)** Estradiol (E_2_).

The data for reproductive hormones FSH, LH, PRL, and progesterone were insufficient in the included studies. Therefore, we did not present forest plots. Two studies reported FSH, LH, and PRL (18, 19), and both included participants with hyperuricemia; two studies reported progesterone (17, 18), with Li et al. reporting data for the gout group. For FSH, both studies showed a decreasing trend in the hyperuricemia group compared with the control group (MD = −0.58, 95% CI = −1.12 to −0.04, *P* = 0.04). For LH and PRL, no significant differences were observed between the hyperuricemia group and the control group (LH: MD = −0.01, 95% CI = −1.05 to 1.03, *P* = 0.98; PRL: MD = 0.04, 95% CI = −1.35 to 1.43, *P* = 0.96). No significant difference was observed in the progestin levels between the gout/hyperuricemia group and the normal uric acid control group (Prog: MD = −0.03, 95% CI = −0.14 to 0.09, *P* = 0.65).

Among the included studies, only one study (enrolling patients with hyperuricemia) (19) reported data related to sperm quality. In this study, the hyperuricemia group exhibited lower semen volume (SV) and total sperm count (TSC) compared to the control group (SV: (2.88 ± 1.32) ml, (3.11 ± 1.27) ml, *P* < 0.05; TSC: (192.85 ± 139.74) × 10^6^, (222.13 ± 153.61) × 10^6^, *P* < 0.05).

### 3.4 Sensitivity analysis

We conducted sensitivity analyses for ED and T data to assess the reliability of our analyses. We recalculated the pooled RR/MD by eliminating one study at a time and generating sensitivity analysis plots using Stata 18.0. The results presented a similar trend, with no single study exerting a significant influence on the overall effect estimate. The RR range for ED was 1.32 (95% CI = 1.14 to 1.52) to 1.45 (95% CI = 1.30 to 1.62) after excluding one study at a time (**Figure 4**; **Supplementary Table 4**). The MD range for T was −1.11 (95% CI = −1.49 to −0.72) to −0.91 (95% CI = −1.24 to −0.58) after excluding one study at a time (**Figure 5**; **Supplementary Table 5**).

**Figure 4:**
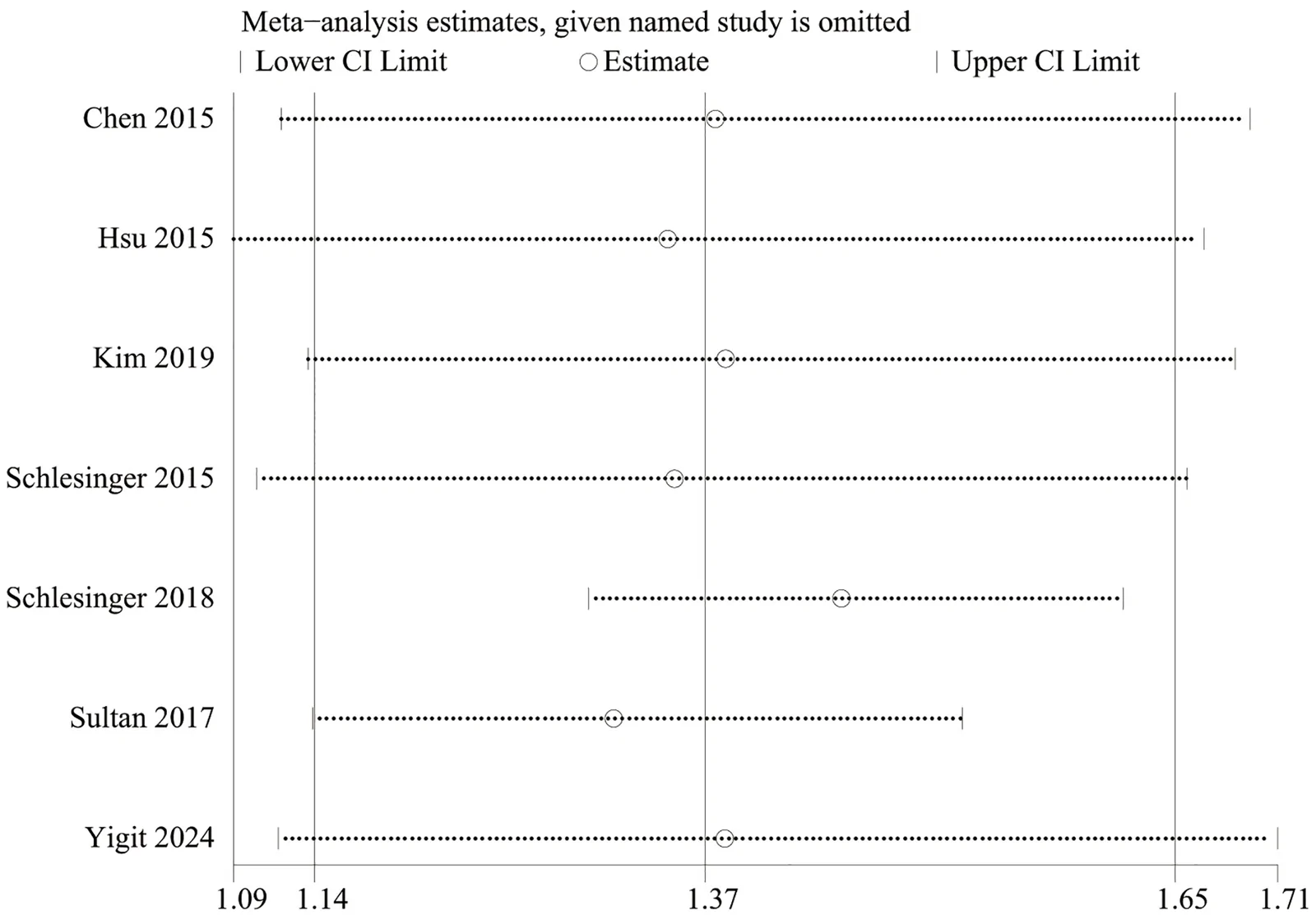
Leave-one-out sensitivity analysis of the association between gout and ED.

**Figure 5:**
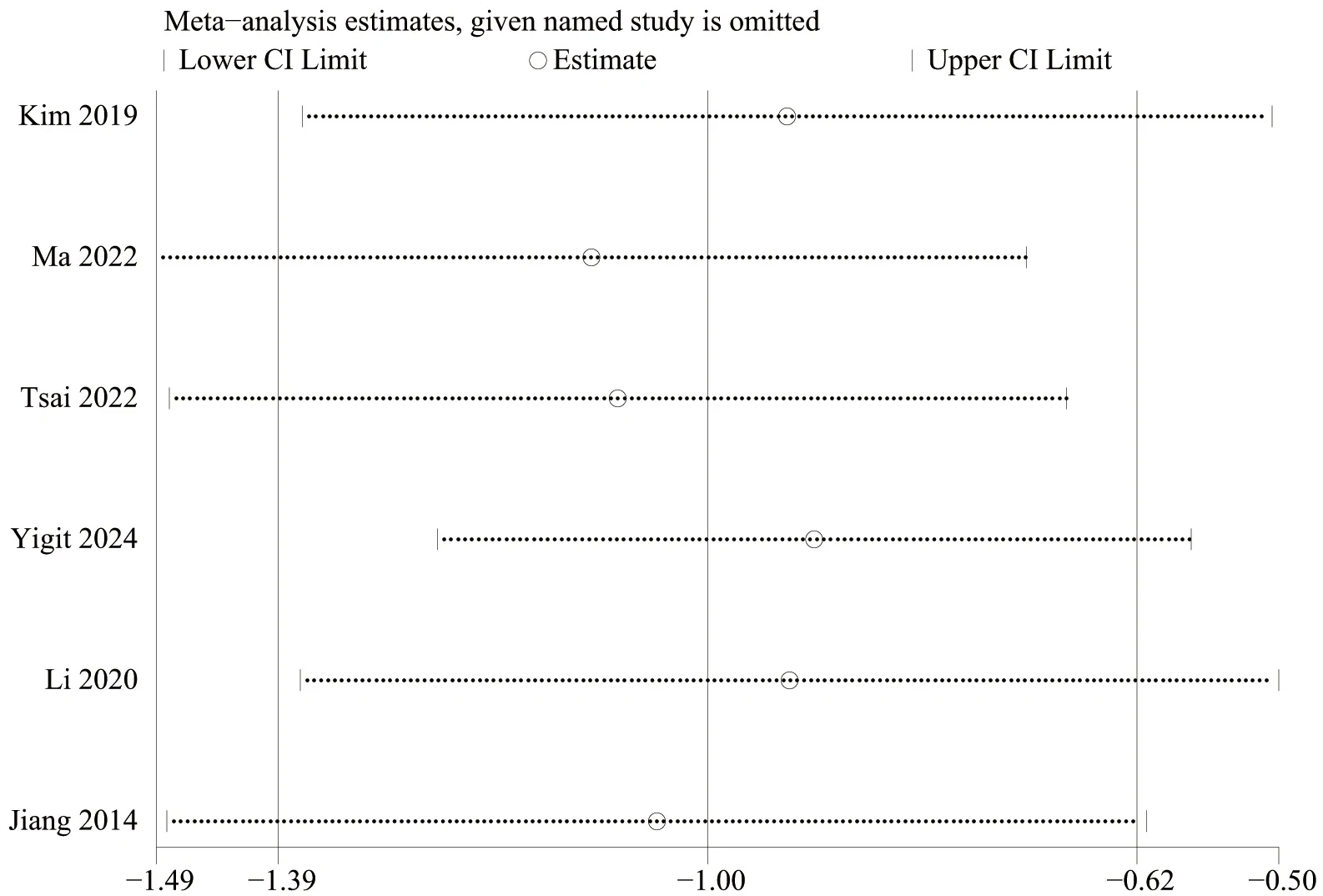
Leave-one-out sensitivity analysis of the association between gout or hyperuricemia and testosterone.

### 3.5 Subgroup analysis

To further assess the relationship of gout and hyperuricemia with male reproductive health, we performed subgroup analyses of data on ED according to age, region, study type, and evaluation criteria for ED and gout. Age groups (≤34, 35–64, and ≥65 years) and inflammatory subgroups (defined as clinical gout versus asymptomatic hyperuricemia) were pre-specified (see Methods). The results are shown in **Table 2**. For data related to the T hormone, we conducted subgroup analysis according to region, study type, and the presence or absence of inflammation, and the specific results are shown in **Table 3**.

**Table 2:** Subgroup analysis of the association between gout and ED risk.

Study or subgroup	No. of studies	Heterogeneity		RR (95% CI)	*P*
		I^2^ (%)	*P*		
Age (years)					
≤ 34	3	0	0.42	1.99(1.55, 2.55)	0.00
35-64	3	74	0.02	1.51(1.32, 1.73)	0.00
≥ 65	1	-	-	1.39(1.07, 1.81)	0.01
Area					
Asian	3	0	0.47	1.43(1.31, 1.57)	0.00
Non-Asian	4	98	0.00	1.37(1.04, 1.79)	0.02
Study design					
Cohort study	4	98	0.00	1.38(1.06, 1.79)	0.02
Cross-sectional study	3	0	0.55	1.35(1.21, 1.50)	0.00
ED assessment criteria					
IIEF	3	0	0.53	1.34(1.21, 1.49)	0.00
ICD - codes	2	24	0.25	1.44(1.28, 1.61)	0.00
Read codes	1	-	-	1.09(1.04, 1.14)	0.00
medical codes by physician	1	-	-	1.67(1.58, 1.75)	0.00
Gout assessment criteria					
ACR	3	0	0.53	1.34(1.21, 1.49)	0.00
ICD - codes	2	24	0.25	1.44(1.28, 1.61)	0.00
Read codes	2	99	0.00	1.37(1.14, 1.65)	0.16

ED, erectile dysfunction; RR, relative risk; CI, confidence interval.

**Table 3:** Subgroup analysis of the association between gout or hyperuricemia and T levels.

Study or subgroup	No. of studies	Heterogeneity		MD (95% CI)	*P*
		I^2^ (%)	*P*		
Area					
Asian	5	84	0.00	-0.91(-1.24, -0.58)	0.00
Non-Asian	1	-	-	-1.38(-1.47, -1.29)	0.00
Study design					
Cohort study	1	-	-	-0.63(-0.82, -0.44)	0.00
Cross-sectional study	5	92	0.00	-1.08(-1.48, -0.69)	0.00
Inflammatory reaction					
Yes	3	0	0.99	-1.38(-1.47, -1.30)	0.00
No	3	0	0.37	-0.62(-0.76, -0.49)	0.00

T, testosterone; MD, mean difference; CI, confidence interval.

In ED-related subgroup analyses, similar associations were observed across age, region, study type, and ED assessment criteria. When stratified by gout diagnostic criteria, a statistically significant association between gout and ED risk was found in groups using ACR and ICD codes, whereas no association was observed in the Read codes group. Specific subgroup analysis forest plots of ED are shown in **Supplementary Images 1–5**.

In the T-related subgroup analysis stratified by inflammation status, the inflammatory group showed a MD of −1.38 (95% CI = −1.14 to −1.30, *P* < 0.001), whereas the non-inflammatory group showed an MD of −0.62 (95% CI = −0.76 to −0.49, *P* < 0.001). Both subgroups showed *P* < 0.0001, with I² = 0% and Tau² = 0.00, thereby indicating complete homogeneity with no between-study variation. The presence or absence of inflammation explained 98.8% of the between-subgroup variation, suggesting that inflammation was a strong effect modifier. Forest plots for the T subgroup analyses are shown in **Supplementary Images 6**.

### 3.6 Publication bias

Both the Begg and Egger tests for ED indicated no significant publication bias (Begg test, *P* > |z| = 0.548; Egger test, *P* > |t| = 0.731). For T, both the Begg and Egger tests indicated no significant publication bias (Begg test, *P* > |z| = 0.133; Egger test, *P* > |t| = 0.281). The specific inspection diagrams are shown in **Supplementary Images 7**, **8**.

The power of these tests was limited because of the small number of included studies (less than 10 studies for each outcome). Therefore, nonsignificant results should be interpreted with caution (see Discussion for more details).

## 4 Discussion

### 4.1 Interpretation and implications

Clinicians often overlook the reproductive health of patients when managing gout, and most men with gout are not aware of the potential risk of infertility. This reflects limited awareness among physicians and patients regarding the association between gout and fertility, and the fact that reproductive health issues have received less attention in clinical practice. Since the prevalence of gout has increased in recent years, more patients have expressed concern about fertility. This has prompted clinicians to adopt more rational and comprehensive treatment strategies for gout, with greater attention to reproductive health in order to better preserve and protect fertility.

This meta-analysis showed that the prevalence of ED among male patients with gout was higher than among normouricemic men (**Figure 2**). Because of significant heterogeneity among the included studies, we performed sensitivity and subgroup analyses. In the subgroup analyses, the lowest RR for ED was 1.09 (95% CI = 1.04 to 1.14) in a population-based, BMI-matched study by Schlesinger et al. The mean age of the study participants was relatively high, 63.6 ± 12.3 years in the gout group and 63.6 ± 12.2 years in the control group (33). The higher rate of ED underreporting among older men may partly explain the lower RR in that study. Subgroup analyses stratified by diagnostic criteria for gout and ED better accounted for the heterogeneity. This suggests that differences in diagnostic criteria may influence study findings.

Our findings may be explained by two non-mutually exclusive mechanisms.

On one hand, gout may directly contribute to ED through several biological pathways. Endothelial dysfunction is central to the pathogenesis of ED. Hyperuricemia promotes endothelial dysfunction through multiple mechanisms. It stimulates proliferation of vascular smooth muscle cells, activates the renin-angiotensin system, and reduces nitric oxide (NO) bioavailability by increasing oxidative stress and impairing endothelial NO synthase activity. These changes impair vasodilation and ultimately lead to ED (36).

Inflammation and immune dysregulation may further amplify the impact of gout on ED. Monosodium urate (MSU) crystal deposition activates the NLRP3 inflammasome and releases pro-inflammatory cytokines (especially IL-6, IL-1β, and TNF-α). IL-6 downregulates endothelial NO synthase and promotes endothelial apoptosis, whereas TNF-α induces insulin resistance (IR) in the vascular endothelium, thereby further impairing NO production (37). These mechanisms not only affect systemic vascular function but may also impair gonadal function, as discussed in detail below.

Furthermore, gout is a form of inflammatory arthritis that is associated with persistent low-grade inflammation and may lead to cumulative endothelial damage over time (36, 38). This may also help explain the increased risk of ED observed across different age groups in our subgroup analysis. Beyond physical mechanisms, the psychological burden of gout may also adversely affect sexual health. Patients with gout have higher rates of depression, anxiety, and fatigue, all of which are independent risk factors for ED (39).

On the other hand, gout often coexists with a broader cardiac metabolic burden, including obesity, IR, hypertension, and dyslipidemia, and may serve as a clinical marker for these conditions. These comorbidities are recognized as independent risk factors for ED and endocrine dysfunction (20, 22, 38). They promote endothelial dysfunction through overlapping inflammatory and oxidative pathways, reduce NO bioavailability, and impair testosterone synthesis (40). Because of the observational nature of the included studies, our meta-analysis cannot distinguish the direct effect of gout from the combined effects of its associated comorbidities.

Decreased male fertility is partly attributed to reproductive hormone deficiencies resulting from hypogonadism. Testosterone is the major male reproductive hormone and is essential for the maintenance fertility. Epidemiological studies have shown that gout predominantly affects men over 40 years of age and postmenopausal women, with marked sex differences in prevalence, thereby suggesting a potential close association between reproductive hormones and gout (17). Our meta-analysis primarily collected data on T and E_2_ levels. The results showed that T and E_2_ levels were lower in male patients with gout or hyperuricemia than in the normouricemic men (**Figure 3**). Both analyses of T and E_2_ showed high heterogeneity.

A subgroup analysis of six studies reporting T levels (**Table 3**) showed that stratification by the presence or absence of an inflammatory response effectively explained the source of heterogeneity. The effect size in the inflammatory group (i.e., the gout group) was approximately 2.2 times that in the non-inflammatory group (i.e., the hyperuricemia group). Three studies reported estradiol levels, but also showed high heterogeneity (I^2^ = 91%). This was likely because of the limited number of studies. Among these three studies, the mean age of participants in the study by Li et al. (17) was significantly higher than in the studies by Jiang et al. (18) and Ma et al. (19). Furthermore, Li et al. included male patients with gout, whereas the other two studies enrolled male subjects with hyperuricemia. These factors may account for the observed heterogeneity.

The mechanisms underlying reproductive hormone alterations in gout and hyperuricemia are multifactorial. Testosterone exerts anti-inflammatory effects in tissues and organs by reducing the release of pro-inflammatory cytokines such as TNF-α, IL-6, and IL-1β (41). These cytokines have been shown to inhibit the HPG axis, reduce the secretion of gonadotropins (FSH, LH), and directly inhibit steroid synthesis in Leydig cells, thereby reducing the secretion of T and E_2_ (42). This is consistent with our observation that testosterone reduction was significantly more pronounced in patients with gout (inflammatory group: MD = −1.38) than in individuals with asymptomatic hyperuricemia (non-inflammatory group: MD = −0.62). This suggested that the inflammatory burden in gout amplifies the suppressive effect on testosterone beyond hyperuricemia alone. Concurrently, urate crystal deposition in gout causes oxidative damage. This impairs testicular function, leading to testicular hypofunction and decreased testosterone levels.

The inflammatory environment in gout not only inhibits Leydig cell steroidogenesis but also affects the production of sex hormone-binding globulin (SHBG) (43). This would further complicate the interpretation of total testosterone levels and amplifies functional androgen deficiency by reducing free testosterone bioavailability.

Beyond inflammation, hyperuricemia itself may contribute to testosterone suppression through distinct mechanisms. Potential mechanisms include IR, reduced renal urate excretion, and systemic protein metabolism alterations that increase purine production (21, 44, 45). Elevated serum uric acid induces oxidative stress in testicular tissue and Leydig cells (46), impairs mitochondrial function, and promotes endothelial dysfunction, all of which may impair steroidogenesis. This metabolic pathway could explain the modest but significant testosterone reduction observed in the hyperuricemia group without clinical gout.

In addition, E_2_ plays a protective role in uric acid metabolism by enhancing insulin sensitivity and promoting renal uric acid excretion (47). Thus, low E_2_ levels in patients with gout or hyperuricemia may generate the following vicious cycle: low E_2_ levels impair urate clearance and increase serum uric acid, whereas inflammation and oxidative stress continue to suppress reproductive hormone production. The interplay between inflammation, hyperuricemia, and reproductive hormones is complex and bidirectional and requires further investigation.

In addition to the meta-analysis results discussed above, a single study conducted among infertile men with hyperuricemia (19) reported that SV and TSC were lower in men with hyperuricemia-related infertility than in the control group with normal uric acid-related infertility. A correlation analysis demonstrated a negative relationship between semen parameters and serum uric acid levels in men (48). Uric acid is an antioxidant in seminal plasma, and its level is significantly reduced in the seminal plasma of men with hyperuricemia. Therefore, increased oxidative stress in the seminal plasma of men with hyperuricemia damages sperm axons, causes abnormal sperm morphology, reduces sperm motility, affects epididymal secretory function, and alters the semen microenvironment (19, 23). However, the study by Ma et al. was a single cross-sectional study of infertile men with hyperuricemia, and its findings cannot be generalized to the broader population with gout or hyperuricemia. Furthermore, the cross-sectional design precludes any causal inference. Thus, the effects of gout and hyperuricemia on semen quality and male fertility remain largely unknown and require further investigation.

Although some clinicians recommend stopping ULT during the reproductive period (49), a prospective cohort study of 49 patients with gout aged 18 to 45 years found that allopurinol and febuxostat did not have clinically relevant negative effects on sperm quality or reproductive hormones over more than 3 months, and benzbromarone even increased sperm concentration (50). However, another study showed a higher risk of ED with febuxostat than with allopurinol (51). These findings are not entirely contradictory, as they involve different outcomes: spermatogenesis and erectile function. Because of the limited and partially conflicting evidence, clinicians must decide individually whether to continue or adjust ULT by weighing disease control against potential drug risks.

Conversely, impaired reproductive health may serve as an early marker of future cardiometabolic disorders. Infertile men exhibit a higher prevalence of hyperuricemia and other comorbidities than the fertile controls (52, 53). Although direct evidence for a bidirectional relationship with gout is limited, they share common pathways, including oxidative stress, IR, and systemic inflammation. Future studies should explore whether reproductive dysfunction predicts an increased risk of gout, which would strengthen the clinical value of comprehensive health screening in infertile men.

Exploring the association between reproductive health and gout or hyperuricemia may represent a plausible strategy to improve both conditions and quality of life, with sexual health serving to enhance treatment adherence. Currently, ED is the primary factor affecting reproductive health in male patients with gout. However, the relationship between gout, hyperuricemia, and male reproductive health is complex, and many biological mechanisms remain to be clarified. Sexual function, reproductive hormones, and sperm quality are also interlinked with factors related to reproductive health. This complexity makes it challenging to study or evaluate any single factor in isolation.

### 4.2 Strengths and limitations

This review presents a study of reproductive health in men with gout or hyperuricemia and demonstrates statistically significant abnormalities through meta-analysis. According to the NOS scale and the AHRQ checklist, low quality studies were not included in this review. Therefore, the results of this review are valuable as a reference.

However, there are a few limitations in this review. First, all included studies were observational in design (cohort or cross-sectional), which is appropriate for examining epidemiological associations but cannot establish causality. The high prevalence of coexisting conditions among gout, hyperuricemia, and cardiometabolic diseases such as obesity, hypertension, and diabetes may partially explain the observed associations, and causality cannot be inferred from our data.

Second, hormonal data were restricted to total testosterone, with no included studies reporting SHBG or calculated free testosterone. This limits the clinical interpretability of our findings because free testosterone is a more sensitive marker of androgen status in patients with chronic inflammation and metabolic disorders (54). Changes in SHBG due to obesity, inflammation, or medications may dissociate total testosterone from free testosterone, and normal or low-normal total testosterone levels could mask a clinically significant free testosterone deficit (55).

Third, fertility potential is a complex concept that cannot be adequately assessed by reproductive hormones or conventional semen parameters alone. A comprehensive andrological evaluation includes testicular ultrasonography, sperm DNA fragmentation testing, and evaluation of clinical outcomes such as time-to-pregnancy, together with SHBG and calculated free testosterone measurements (56, 57). None of the studies in our meta-analysis provided such a comprehensive assessment. Therefore, our findings on fertility should be considered preliminary.

Fourth, the number of studies available for each outcome was small (seven for ED, six for T, and only one for semen parameters). This limits our ability to perform detailed subgroup analyses and may affect the generalizability of our findings to broader populations.

Fifth, Begg’s and Egger’s tests did not show any significant publication bias, but the statistical power of these tests was limited because of fewer than 10 studies per outcome (58). Non-significant results should not be interpreted as strong evidence against publication bias, and small unpublished null studies may still exist.

Furthermore, since most of the included studies did not provide relevant evidence, we did not conduct a comparative analysis between acute and chronic gout.

### 4.3 Future directions

Because of the limitations in the currently available research studies, more high-quality, large-scale, comprehensive Mendelian randomization prospective cohort studies that adjust for confounding factors are needed in the future. Furthermore, investigations are necessary to unravel the underlying mechanisms of the effects of gout and hyperuricemia on male reproductive health and explore the bidirectional relationship between the two. At the same time, future research should include comprehensive male assessments, including SHBG and the calculation of free testosterone values, etc. This will provide a more solid theoretical foundation for clinical practice and provide a basis for formulating targeted intervention strategies, thereby overcoming the existing limitations.

## 5 Conclusions

In summary, this study provides evidence that gout and hyperuricemia are associated with male reproductive health issues, including ED and low reproductive hormones. However, evidence regarding semen quality is limited to a single study in infertile men with hyperuricemia and cannot be generalized. This suggests that clinicians should conduct comprehensive reproductive health assessments when diagnosing and treating male patients with gout or hyperuricemia, especially those of reproductive age, and provide specific interventions when necessary.

## Data Availability

The original contributions presented in the study are included in the article/**Supplementary Material**. Further inquiries can be directed to the corresponding authors.
