# Supplementary material for: Gout, hyperuricemia, and male reproductive health: a systematic review and meta-analysis
Source: Front Immunol. 2026 Sep 15;17:1889720. doi: 10.3389/fimmu.2026.1889720 (PMC13619580; doi:10.3389/fimmu.2026.1889720)
Supplement: Supplementary file 1 [file Supplementaryfile1.pdf]

NIHR | National Institute for Health and Care Research

PROSPERO
International prospective register of systematic reviews

Association Between Gout and Male Reproductive Health: A Systematic Review and Meta-Analysis

Jiping Mao, Dehong Wu

Citation
Jiping Mao, Dehong Wu. Association Between Gout and Male Reproductive Health: A Systematic Review and Meta-Analysis. PROSPERO 2026 CRD420261302205. Available from https://www.crd.york.ac.uk/PROSPERO/view/CRD420261302205.

REVIEW TITLE AND BASIC DETAILS

Review title
Association Between Gout and Male Reproductive Health: A Systematic Review and Meta-Analysis

Condition or domain being studied
Gout; Infertility; Erectile Dysfunction; Hyperuricemia

Rationale for the review
Due to social, family and personal physiological and psychological needs, young and middle-aged men of childbearing age with gout are particularly concerned about whether gout will affect their fertility during medical visits. Rheumatologists need to pay attention to the reproductive health of male patients of childbearing age with gout in clinical treatment. This systematic review and meta-analysis aims to consolidate the current evidence on the impact of gout on male sexual dysfunction, fertility and abnormal reproductive hormones. By organizing and elaborating on the relevant aspects of male reproductive health, we hope to provide valuable suggestions for clinicians in the treatment and management of male patients of childbearing age with gout.

Review objectives
This systematic review and meta-analysis aims to consolidate the current evidence on the impact of gout on male sexual dysfunction, fertility and abnormal reproductive hormones. By organizing and elaborating on the relevant aspects of male reproductive health, we hope to provide valuable suggestions for clinicians in the treatment and management of male patients of childbearing age with gout.

Keywords
Gout; Hyperuricemia; Reproductive health; Erectile dysfunction; Sex hormones

Country
China

ELIGIBILITY CRITERIA

Population

Included
Male patients diagnosed with gout

Intervention(s) or exposure(s)

Included
Hyperuricemia, gout, inflammatory arthritis

Comparator(s) or control(s)

Included
healthy males

Study design
Only nonrandomized study types will be included.

Context
We will compare the incidence of erectile dysfunction (ED), the levels of reproductive hormones and sperm quality between male gout patients and healthy men to verify whether gout has an impact on male reproductive health.

TIMELINE OF THE REVIEW

Date of first submission to PROSPERO
05 February 2026

Review timeline
Start date: 5 February 2026. End date: 5 February 2027.

Date of registration in PROSPERO
05 February 2026

AVAILABILITY OF FULL PROTOCOL

Availability of full protocol
A full protocol has not been written.

SEARCHING AND SCREENING

Search for unpublished studies
Both published and unpublished studies will be sought.

Main bibliographic databases that will be searched

The main databases to be searched are *CLIB - The Cochrane Library*, *Embase - Embase via Ovid*, *Embase.com*, *MEDLINE*, *PubMed* and *SCI - Science Citation Index*.

Search language restrictions

There are no language restrictions.

Search date restrictions

Databases will be searched for articles published from 1 January 2000 and before by 31 October 2025.

Other methods of identifying studies

Other studies will be identified by: *contacting authors or experts*, *looking through all the articles that cite the papers included in the review ("snowballing" or forward citation searching)*, *reference list checking (backward citation searching)*, *searching conference proceedings*, *searching dissertation and thesis databases* and *searching trial or study registers*.

Link to search strategy

A full search strategy is not available.

Selection process

Studies will be screened independently by at least two people (or person/machine combination) with a process to resolve differences.

Other relevant information about searching and screening

None

DATA COLLECTION PROCESS

Data extraction from published articles and reports

Data will be extracted independently by at least two people (or person/machine combination) with a process to resolve differences.

Authors will be asked to provide any required data not available in published reports.

Study risk of bias or quality assessment

Risk of bias will be assessed using: *Newcastle-Ottawa* and *ROBINS-I*

Data will be assessed independently by at least two people (or person/machine combination) with a process to resolve differences.

Additional information will be sought from study investigators if required information is unclear or unavailable in the study publications/reports.

Reporting bias assessment

Risk of bias due to missing results will be assessed

Certainty assessment

We will conduct a final evidence recommendation for all included literature through the Grade evidence grading

OUTCOMES TO BE ANALYSED

Main outcomes

We mainly evaluated from the following three aspects: (1) Sexual function (sexual dysfunction (SD), erectile dysfunction (ED)); (2) Reproductive hormones; (3) Fertility (sperm quality, sperm count, etc.)

Additional outcomes

There are no additional outcomes.

PLANNED DATA SYNTHESIS

Strategy for data synthesis

We used Review Manager 5.4 software to perform meta-analyses of the results for which sufficient data were available (more than two studies provided valid data). RR/OR and 95%CI were used to present the results for binary outcomes and mean difference (MD) for continuous outcomes. When there was a significant statistical difference , the random effects model was used, otherwise the fixed effects model was used. Sensitivity and subgroup analyses were applied to explore the origin of homogeneity. In addition, publication bias was carried out by funnel plots. A systematic review was performed for results with insufficient data. Begg and Egger tests were performed using Stata 18.0 to check for publication bias.

CURRENT REVIEW STAGE

Stage of the review at this submission

Review stage	Started	Completed
Pilot work		
Formal searching/study identification		
Screening search results against inclusion criteria		
Data extraction or receipt of IPD		
Risk of bias/quality assessment		
Data synthesis		

Review status

The review is currently planned or ongoing.

Publication of review results

Results of the review will be published.

REVIEW AFFILIATION, FUNDING AND PEER REVIEW

Review team members

Dr Jiping Mao (review guarantor and contact) ORCID: 0009-0003-3493-0986. The Second Affiliated Hospital of Zhejiang Chinese Medical University. China.

No conflict of interest declared.

Dr Dehong Wu. The Second Affiliated Hospital of Zhejiang Chinese Medical University. China.

No conflict of interest declared.

Named contact

Dr Jiping Mao (maojp831@163.com). ORCID: 0009-0003-3493-0986. The Second Affiliated Hospital of Zhejiang Chinese Medical University. China.

Review affiliation

The Second Affiliated Hospital of Zhejiang Chinese Medical University

Funding source

Review has no funding and no agreed support from an academic institution and is done in authors' own time.

Peer review

There has been no peer review of this planned review.

ADDITIONAL INFORMATION

Review conflict of interest

Declared individual interests are recorded under team member details.. No additional interests are recorded for this review.

Medical Subject Headings

Fertility; Gout; Hormones; Humans; Hyperuricemia; Male; Reproductive Health

SIMILAR REVIEWS

Check for similar records already in PROSPERO

PROSPERO identified a number of existing PROSPERO records that were similar to this one (last check made on 5 February 2026). These are shown below along with the reasons given by that the review team for the reviews being different and/or proceeding.

- Diabetes-induced hyperglycemia impairs male reproductive function: a systematic review [published 22 June 2017] [CRD42017070203]. The review was judged **not to be similar**
- Association between body mass index and male reproductive hormones: a systematic review and meta-analysis [published 4 November 2024] [CRD42024605128]. The review was judged **not to be similar**
- Effects of sleep and circadian rhythm changes on male reproductive health: a systematic review and meta-analysis [published 2 April 2022] [CRD42022312693]. The review was judged **not to be similar**

PROSPERO version history

- Version 1.0, published 05 Feb 2026

Disclaimer

The content of this record displays the information provided by the review team. PROSPERO does not peer review registration records or endorse their content.

PROSPERO accepts and posts the information provided in good faith; responsibility for record content rests with the review team. The guarantor for this record has affirmed that the information provided is truthful and that they understand that deliberate provision of inaccurate information may be construed as scientific misconduct.

PROSPERO does not accept any liability for the content provided in this record or for its use. Readers use the information provided in this record at their own risk.

Any enquiries about the record should be referred to the named review contact
