# Supplementary material for: Gout, hyperuricemia, and male reproductive health: a systematic review and meta-analysis
Source: Front Immunol. 2026 Sep 15;17:1889720. doi: 10.3389/fimmu.2026.1889720 (PMC13619580; doi:10.3389/fimmu.2026.1889720)
Supplement: Supplementary file 2 [file Supplementaryfile2.pdf]

Databases	Search terms	Numbers of records
**Pubmed**	Search: (("Gout"[Mesh]) OR (((((((((Gout[Title/Abstract]) OR (Gouty Arthritis[Title/Abstract])) OR (Urate Crystal Arthritis[Title/Abstract])) OR (Crystal Arthropathy, Urate[Title/Abstract])) OR (Monosodium Urate Crystal Deposition Disease[Title/Abstract])) OR (Hyperuricemia[Title/Abstract])) OR (Tophus[Title/Abstract])) OR (Tophi[Title/Abstract])) OR (Podagra[Title/Abstract]))) AND (((((((((((((((("Infertility, Male"[MeSH]) OR (Male Infertility[Title/Abstract])) OR ("Sterility, Male"[Title/Abstract])) OR (Male Sterility[Title/Abstract])) OR ("Subfertility, Male"[Title/Abstract])) OR (Male Subfertility[Title/Abstract])) OR ("Sub-Fertility, Male"[Title/Abstract])) OR (Male Sub-Fertility[Title/Abstract])) OR ("Sub Fertility, Male"[Title/Abstract])) OR (Aspermia[Title/Abstract])) OR (Asthenozoospermia[Title/Abstract])) OR (Azoospermia[Title/Abstract])) OR (Oligospermia[Title/Abstract])) OR (Sertoli Cell-Only Syndrome[Title/Abstract])) OR (Teratozoospermia[Title/Abstract])) OR (((((((("Spermatozoa"[MeSH])) OR (Spermatozoon[Title/Abstract])) OR (Sperm[Title/Abstract])) OR (Acrosome[Title/Abstract])) OR (Spermatids[Title/Abstract])) OR (Spermatocytes[Title/Abstract])) OR (Spermatogonia[Title/Abstract])) OR (((Semen[Title/Abstract]) OR (Semen analysis[Title/Abstract]))))) OR (("Male"[Mesh]) AND ((((((((Reproductive system[Title/Abstract]) OR (Reproductive[Title/Abstract])) OR ("Hormones"[Mesh])) OR ("Testosterone"[Mesh])) OR ("Progesterone"[Mesh])) OR ("Prolactin"[Mesh])) OR ("Estradiol"[Mesh])) OR ("Luteinizing Hormone"[Mesh])) OR ("Follicle Stimulating Hormone"[Mesh])))) OR ((Erectile dysfunction[Title/Abstract]) OR ((Sexual dysfunction[Title/Abstract]) AND (male[Title/Abstract]))))	414

**Cochrane**	#1 MeSH descriptor: [Gout] explode all trees<br>#2 (Gout):ti,ab,kw OR (Gouty Arthritis):ti,ab,kw OR (Urate Crystal Arthritis):ti,ab,kw OR (Crystal Arthropathy, Urate):ti,ab,kw OR (Monosodium Urate Crystal Deposition Disease):ti,ab,kw OR (Hyperuricemia):ti,ab,kw OR (Tophus):ti,ab,kw OR (Tophi):ti,ab,kw OR (Podagra):ti,ab,kw<br>#3 #1 OR #2<br>#4 MeSH descriptor: [Infertility,Male] explode all trees<br>#5 (Male Infertility):ti,ab,kw OR (Sterility, Male):ti,ab,kw OR (Male Sterility):ti,ab,kw OR (Subfertility, Male):ti,ab,kw OR (Male Subfertility):ti,ab,kw OR (Sub-fertility, Male):ti,ab,kw OR (Male Sub-fertility):ti,ab,kw OR (Sub fertility, Male):ti,ab,kw OR (Aspermia):ti,ab,kw OR (Asthenozoospermia):ti,ab,kw OR (Azoospermia):ti,ab,kw OR (Oligospermia):ti,ab,kw OR (Sertoli Cell-only Syndrome):ti,ab,kw OR (Asthenozoospermia):ti,ab,kw<br>#6 #4 OR #5<br>#7 MeSH descriptor: [Spermatozoa] explode all trees<br>#8 (Spermatozoon):ti,ab,kw OR (Sperm):ti,ab,kw OR (Acrosome):ti,ab,kw OR (Spermatids):ti,ab,kw OR (Spermatocytes):ti,ab,kw OR (Spermatogonia):ti,ab,kw OR (semen):ti,ab,kw OR (semen analysis):ti,ab,kw<br>#9 #7 OR #8<br>#10 MeSH descriptor: [Hormones] explode all trees<br>#11 MeSH descriptor: [Testosterone] explode all trees<br>#12 MeSH descriptor: [Progesterone] explode all trees<br>#13 MeSH descriptor: [Prolactin] explode all trees<br>#14 MeSH descriptor: [Estradiol] explode all trees<br>#15 MeSH descriptor: [Luteinizing Hormone] explode all trees<br>#16 MeSH descriptor: [Follicle Stimulating Hormone] explode all trees<br>#17 #10 OR #11 OR #12 OR #13 OR #14 OR #15 OR #16<br>#18 (Reproductive):ti,ab,kw OR (Reproductive System):ti,ab,kw OR (Sexual function):ti,ab,kw<br>#19 #17 OR #18<br>#20 MeSH descriptor: [Male] in all MeSH products<br>#21 #19 AND #20<br>#22 #6 OR #9 OR #17 OR #21<br>#23 #3 AND #22	74
**Embase**	#1 'gout'/exp OR 'gout':ti,ab,kw OR 'gouty arthritis':ti,ab,kw OR 'urate crystal arthritis':ti,ab,kw OR 'crystal arthropathy, urate':ti,ab,kw OR 'monosodium urate crystal deposition disease':ti,ab,kw OR 'hyperuricemia':ti,ab,kw OR 'tophus':ti,ab,kw OR 'tophi':ti,ab,kw OR 'podagra':ti,ab,kw	280

	#2 'male infertility'/exp OR 'infertility, male':ti,ab,kw OR 'male steritility':ti,ab,kw OR 'steritility, male':ti,ab,kw OR 'subfertility, male':ti,ab,kw OR 'male subfertility':ti,ab,kw OR 'sub-fertility, male':ti,ab,kw OR 'male sub-fertility':ti,ab,kw OR 'sub fertility, male':ti,ab,kw OR 'aspermia':ti,ab,kw OR 'asthenozoospermia':ti,ab,kw OR 'azoospermia':ti,ab,kw OR 'oligospermia':ti,ab,kw OR 'sertolicell-only syndrome':ti,ab,kw OR 'teratozoospermia':ti,ab,kw<br>#3 'spermatozoon'/exp OR 'spermaticcell':ti,ab,kw OR 'spermatozoa':ti,ab,kw OR 'spermatozoid':ti,ab,kw OR 'semen':ti,ab,kw OR 'semen analysis':ti,ab,kw<br>#4 'hormones'/exp OR 'testosterone'/exp OR 'progesterone'/exp OR 'prolactin'/exp OR 'estradiol'/exp OR 'luteinizing hormone'/exp OR 'reproductive system':ti,ab,kw OR 'reproductive':ti,ab,kw OR 'Sexual function':ti,ab,kw<br>#5 'male'/exp<br>#6 #4 AND #5<br>#7 #2 OR #3 OR #6<br>#8 #1 AND #7	
**Web of science**	#1 TS=("Gout" OR "Gouty Arthritis" OR "Urate Crystal Arthritis" OR "Crystal Arthropathy, Urate" OR "Monosodium Urate Crystal Deposition Disease" OR "Hyperuricemia" OR "Tophus" OR "Tophi" OR "Podagra") and Preprint Citation Index (Exclude – Database)<br>#2 TS=("Infertility, Male" OR "Male Infertility" OR "Sterility, Male" OR "Male Sterility" OR "Subfertility, Male" OR "Male Subfertility" OR "Sub-Fertility, Male" OR "Male Sub-Fertility" OR "Sub Fertility, Male" OR "Aspermia" OR "Asthenozoospermia" OR "Azoospermia" OR "Oligospermia" OR "Sertoli Cell-Only Syndrome" OR "Teratozoospermia") and Preprint Citation Index (Exclude – Database)<br>#3 TS=("Spermatozoa" OR "Spermatozoon" OR "Sperm" OR "Acrosome" OR "Spermatids" OR "Spermatocytes" OR "Spermatogonia" OR "Semen" OR "Semen Analysis") and Preprint Citation Index (Exclude – Database)<br>#4 TS=("Hormones" OR "Testosterone" OR "Progesterone" OR "Prolactin" OR "Estradiol" OR "Luteinizing Hormone" OR "Follicle Stimulating Hormone" OR "Reproductive" OR "Reproductive System") and Preprint Citation Index (Exclude – Database)<br>#5 TS=("Reproductive" OR "Reproductive System" OR "Sexual function") and Preprint Citation Index (Exclude – Database)<br>#6 #4 OR #5	511

	#7 TS=("Male") and Preprint Citation Index (Exclude – Database)<br>#8 #6 AND #7 and Preprint Citation Index (Exclude – Database)<br>#9 #2 OR #3 OR #8 and Preprint Citation Index (Exclude – Database)<br>#10 #9 AND #1 and Preprint Citation Index (Exclude – Database)	
**CNKI**	SU % =(gout OR gouty arthritis OR hyperuricemia OR tophi OR urate) OR (gout AND kidney disease)) AND SU % =(male AND (infertility OR reproduction OR sex hormones OR sexual function)) OR semen OR sperm)	99
**Wang Fang data**	Topic: (Gout OR gouty arthritis OR hyperuricemia OR tophi OR urate OR (gout AND nephropathy)) AND Topic: ((male AND (infertility OR reproduction OR sex hormone OR sexual function)) OR semen OR sperm)	149
**Total**	2000-2025	1527
