# Supplementary material for: Gout, hyperuricemia, and male reproductive health: a systematic review and meta-analysis
Source: Front Immunol. 2026 Sep 15;17:1889720. doi: 10.3389/fimmu.2026.1889720 (PMC13619580; doi:10.3389/fimmu.2026.1889720)
Supplement: Supplementary file 3 [file Table1.docx]

**Supplementary Table 1** Associations Between Gout/Hyperuricemia and Outcomes.

| Reference | Case | Outcomes | | | Incidence/Prevalence/Findings |
| --- | --- | --- | --- | --- | --- |
|  |  | Sexual Function | Reproductive Hormones | Fertility |  |
| Chen et al. | 19368/77472 | ED↑ | NS | NS | Incidence of ED in gout patients: 12.36 per 10,000 person-years  Incidence of ED in controls: 9.07 per 10,000 person-years  (*P* < .001) Relative risk: 1.36 |
| Hsu et al. | 35265/70529 | ED↑ | NS | NS | Incidence of ED in gout patients:17.6 per 10,000 person-years  Incidence of ED in controls: 12.1 per 10,000 person-years  (*P*-value not listed) Relative risk: 1.45 |
| Kim et al. | 80/70 | ED↑ | T↓ | NS | Incidence of ED higher in the patient group than in the control group  (55.3% vs. 41.4% in control, *P* = 0.047)  Relative risk: 1.35 |
| Schlesinger et al. | 83/118 | ED↑ | NS | NS | Incidence of ED higher in the patient group than in the control group  (76 % vs. 51% in control, *P* = 0.0003)  Relative risk: 1.49 |
| Schlesinger et al. | 38438/154332 | ED↑ | NS | NS | Incidence of ED in gout patients:11.9 per 1000 person-years  Incidence of ED in controls: 10.5 per 1000 person-years  (*P*-value not listed) Relative risk: 1.13 |
| Sultan et al. | 9653/38218 | ED↑ | NS | NS | Incidence of ED in gout patients: 192.8 per 10,000 person-years  Incidence of ED in controls: 136.2 per 10,000 person-years  (*P*-value not listed) Relative risk: 1.42 |
| Yigit et al. | 134/104 | ED↑ | T↓ | NS | The mean IIEF-EF score of gout patients (21.16 ± 2.87)  The mean IIEF-EF score of controls (23.75 ± 2.43) (*P* < 0.001) |
| Li M. L. et al. | 266/129 | NS | T↓, E_2_↓  Prog↑ | NS | pGA group serum E_2_ and T levels below the HC group  [(30 ± 8) pg/ml, (35 ± 10) pg/ml, *P* < 0.05],  [(4.4 ± 1.6) pg/ml, (5.8 ± 1.9) pg/ml, *P* < 0.05]  HC in serum Prog level below the pGA group  [(0.24 ± 0.10)ng/ml , (0.27 ± 0.11) ng/ml, *P* < 0.05] |
| Jiang Y. et al. | 43/43 | NS | T↓E_2_↓PRL↓  FSH↓, Prog↓ | NS | HUA group T below controls [(3.78 ± 0.63) μg/L, (4.56 ± 0.79) μg/L, *P* < 0.05] |
| Ma J et al. | 263/391 | NS | FSH↓,LH↑  PRL↑,T↓ | SV↓,SC↓  TSC↓,PR↓ | HUA group of SV and TSC below controls  [(2.88 ± 1.32) ml , (3.11 ± 1.27) ml ,*P* < 0.05], [(192.85 ± 139.74), (222.13 ± 153.61), *P* < 0.05]  HUA group of T below controls  [(4.05 ± 1.62) ng/ml, (4.54 ± 1.89) ng/ml, *P* < 0.05]  HUA group of LH is higher than controls [(4.26 ± 2.22) mIU/ml, (3.74 ± 1.83) mIU/ml, *P* < 0.05] |
| Tsai et al. | 684/1215 | NS | T↓ | NS | SU ≥7 mg/dL group of T below SU <7 mg/dL group  [(4.70 ± 2.08) mg/dL, (5.33 ± 2.05) mg/dL, *P* < 0.01] |

NS:Not stated; ↑gout patients had higher values than healthy controls; ↓gout patients had lower values than healthy controls; ED:erectile dysfunction; T:testosterone; E_2_:estradiol; PRL:prolactin; FSH:follicle-stimulating hormone; LH:luteinizing hormone; Prog:progesterone; SV:semen volume; SC:sperm concentration; TSC:total sperm count; PR:progressive motility; HUA:hyperuricemia.
