# Supplementary material for: Gout, hyperuricemia, and male reproductive health: a systematic review and meta-analysis
Source: Front Immunol. 2026 Sep 15;17:1889720. doi: 10.3389/fimmu.2026.1889720 (PMC13619580; doi:10.3389/fimmu.2026.1889720)
Supplement: Supplementary file 4 [file Table2.docx]

**Supplementary Table 2** Newcastle-Ottawa Scale (NOS) assessment of the quality of the cohort studies.

| Study | Selection | | | | Comparability | | Exposure/Outcome | | | Total scores |
| --- | --- | --- | --- | --- | --- | --- | --- | --- | --- | --- |
|  | 1 | 2 | 3 | 4 | 5 | 6 | 7 | 8 | 9 |  |
| Chen et al. 2015 | L | L | L | L | L | H | L | L | H | 7 |
| Hsu et al. 2015 | L | L | L | L | L | H | L | L | L | 8 |
| Sultan et al. 2017 | L | L | L | L | L | L | L | L | L | 9 |
| Schlesinger et al. 2018 | L | L | L | L | L | L | L | L | L | 9 |
| Tsai et al.2022 | L | L | L | L | L | L | L | L | H | 8 |

For cohort studies:

1. indicates that the exposed cohort was representative of the population;

2. Indicates that the non-exposed cohort was drawn from the same population;

3. Indicates that the exposure ascertainment was from secure records or a structured interview;

4. Indicates that outcome of interest was not present at start of study;

5. Indicates that the cohorts were comparable for age and sex;

6. Indicates that the cohorts were comparable on all additional factor(s) reported;

7. Indicates that outcome was assessed from a secure record;

8. Indicates that follow-up was long enough for outcomes to occur;

9. Indicates that follow-up was complete.
