# Supplementary material for: Gout, hyperuricemia, and male reproductive health: a systematic review and meta-analysis
Source: Front Immunol. 2026 Sep 15;17:1889720. doi: 10.3389/fimmu.2026.1889720 (PMC13619580; doi:10.3389/fimmu.2026.1889720)
Supplement: Supplementary file 5 [file Table3.docx]

**Supplementary Table 3**. The methodological quality of the cross-sectional study

| Item | Kim et al.2017 | Ma et al.2022 | Schlesinger et al. 2015 | Yigit et al.2024 | Li M.L. et al.2020 | Jiang Y. et al.2014 |
| --- | --- | --- | --- | --- | --- | --- |
| 1) Define the source of information (survey, record review) | L | L | L | L | L | L |
| 2) List inclusion and exclusion criteria for exposed and unexposed subjects (cases and controls) or refer to previous publications | L | L | L | L | L | L |
| 3) Indicate time period used for identifying patients | L | L | L | L | L | L |
| 4) Indicate whether or not subjects were consecutive if not population-based | L | L | L | L | H | H |
| 5) Indicate if evaluators of subjective components of study were masked to other aspects of the status of the participants | Unclear | Unclear | Unclear | Unclear | Unclear | Unclear |
| 6) Describe any assessments undertaken for quality assurance purposes (e.g., test/retest of primary outcome measurements) | L | L | L | L | L | L |
| 7) Explain any patient exclusions from analysis | L | L | L | L | L | L |
| 8) Describe how confounding was assessed and/or controlled. | L | H | L | L | H | H |
| 9) If applicable, explain how missing data were handled in the analysis | H | H | H | H | H | H |
| 10) Summarize patient response rates and completeness of data collection | L | L | L | L | L | L |
| 11) Clarify what follow-up, if any, was expected and the percentage of patients for which incomplete data or follow-up was obtained | H | H | H | H | H | H |
