# Supplementary material for: Gout, hyperuricemia, and male reproductive health: a systematic review and meta-analysis
Source: Front Immunol. 2026 Sep 15;17:1889720. doi: 10.3389/fimmu.2026.1889720 (PMC13619580; doi:10.3389/fimmu.2026.1889720)
Supplement: Supplementary file 6 [file Table4.docx]

**Supplementary Table 4**. Sensitivity analysis of studies on ED after individual exclusion.

| Study omitted | RR (95% CI) for remainders | Heterogeneity | |
| --- | --- | --- | --- |
|  |  | I^2^ (%) | *P* |
| Chen2015 | 1.38[1.12, 1.70] | 97 | 0.000 |
| Hsu 2015 | 1.35[1.09, 1.67] | 97 | 0.000 |
| Kim 2019 | 1.38[1.13, 1.69] | 97 | 0.000 |
| Schlesinger 2015 | 1.35[1.10, 1.66] | 97 | 0.000 |
| Schlesinger 2018 | 1.45[1.30, 1.62] | 75 | 0.001 |
| Sultan 2017 | 1.32[1.14, 1.52] | 87 | 0.000 |
| Yigit 2024 | 1.38[1.12, 1.71] | 97 | 0.000 |

ED erectile dysfunction, RR relative risk, CI confidence interval
