# Supplementary material for: Gout, hyperuricemia, and male reproductive health: a systematic review and meta-analysis
Source: Front Immunol. 2026 Sep 15;17:1889720. doi: 10.3389/fimmu.2026.1889720 (PMC13619580; doi:10.3389/fimmu.2026.1889720)
Supplement: Supplementary file 7 [file Table5.docx]

**Supplementary Table 5**. Sensitivity analysis of studies on T after individual exclusion.

| Study omitted | MD (95% CI) for remainders | Heterogeneity | |
| --- | --- | --- | --- |
|  |  | I^2^ (%) | *P* |
| Kim 2019 | -0.93[-1.36, -0.50] | 95 | 0.000 |
| Ma 2022 | -1.11[-1.49, -0.72] | 93 | 0.000 |
| Tsai 2022 | -1.08[-1.48, -0.69] | 92 | 0.000 |
| Yigit 2024 | -0.91[-1.24, -0.58] | 84 | 0.000 |
| Li 2020 | -0.93[-1.37, -0.50] | 95 | 0.000 |
| Jiang 2014 | -1.05[-1.48, -0.62] | 95 | 0.000 |

T testosterone, MD mean difference, CI confidence interval
